# Association between inflammatory indicators derived from preconception complete blood count and specific adverse fetal, neonatal, and pregnancy outcomes: a national cohort study

**DOI:** 10.3389/fendo.2026.1886578

**Published:** 2026-07-22

**Authors:** Yue Jiang, Shuaihua Song, Hanze Du, Liyuan Zhang, Shi Chen, Hui Pan

**Affiliations:** Department of Endocrinology, Peking Union Medical College Hospital, Chinese Academy of Medical Sciences & Peking Union Medical College, Translational Medicine Center, State Key Laboratory of Complex Severe and Rare Diseases, National Health Commission Key Laboratory of Endocrinology, Beijing, China

**Keywords:** complete blood count, inflammatory indicators, preconception inflammation, pregnancy complications, specific adverse pregnancy outcomes

## Abstract

**Background:**

Adverse fetal and neonatal outcomes, including small for gestational age (SGA), large for gestational age (LGA), and low birth weight (LBW), seriously endanger maternal and infant health. Currently, the associations between preconception baseline immune-inflammatory status and the above specific adverse pregnancy outcomes still lack systematic population-based cohort evidence.

**Methods:**

Based on the National Free Preconception Health Examination Program in China from 2010 to 2012, this large-sample cohort study recruited rural women of childbearing age in China. Multivariate logistic regression and restricted cubic spline (RCS) models were used to systematically analyze the associations between preconception complete blood count (CBC)-derived inflammatory indicators, including neutrophil-to-lymphocyte ratio (NLR), platelet-to-lymphocyte ratio (PLR), systemic immune-inflammation index (SII), and adverse fetal, neonatal, and pregnancy outcomes.

**Results:**

A total of 49,855 preconception participants were enrolled in this study, among whom 16,105 cases experienced adverse fetal and neonatal outcomes. Quartile analysis revealed distinct outcome-specific associations of inflammatory indicators. Moderate levels of NLR increased the risk of SGA, while moderate NLR and SII levels reduced the risk of LGA; the highest quartile of PLR elevated the risk of LGA, and moderate NLR decreased the risk of preterm birth. Only high levels of NLR exerted a protective effect against HBW. Further RCS analyses confirmed significant U-shaped nonlinear associations of NLR, PLR, and SII with LGA, as well as U-shaped correlations of PLR and SII with preterm birth, indicating obvious threshold effects of preconception inflammation on pregnancy outcomes.

**Conclusion:**

After adjusting available covariates, preconception NLR, PLR, and SII exhibited outcome-specific threshold associations with adverse fetal and neonatal outcomes, yet unmeasured confounders (preconception infection, maternal nutrition, etc.) may overestimate these correlations. Owing to the large sample, many significant results carry modest effect sizes with limited individual clinical utility; these markers merely offer population-based epidemiological references instead of standalone screening indicators for rural preconception risk stratification. Our results require validation in modern urban and multicenter cohorts due to the historical rural-only study population.

## Background

Pregnancy outcomes are closely associated with maternal and infant health. Adverse pregnancy outcomes, including preterm birth, fetal growth restriction, low birth weight (LBW), and large for gestational age (LGA), not only cause immediate health threats to mothers and offspring but also increase the risk of long-term chronic complications, thereby adversely affecting lifelong maternal and infant health (1–3). Inflammation is a fundamental biological process throughout gestation and exerts dual regulatory effects on pregnancy progression. Moderate physiological inflammation facilitates normal placental development and maintains uncomplicated pregnancy progression, whereas excessive or dysregulated inflammatory responses contribute substantially to the occurrence of multiple adverse pregnancy complications, including preeclampsia, placental insufficiency, and preterm birth (4–6). At present, conventional inflammatory biomarkers, such as C-reactive protein, interleukin-6, and tumor necrosis factor-α, are widely applied in evaluating systemic inflammatory status and exploring inflammation-related pregnancy disorders (7, 8). However, these indicators are limited by high detection costs and complicated testing procedures, making them infeasible for large-scale population screening and long-term dynamic monitoring in preconception populations. Therefore, it is imperative to identify economical, accessible, and efficient inflammatory markers for preconception risk assessment. Complete blood count (CBC) is a routine, readily available clinical test that evaluates peripheral blood cell components and provides essential baseline clinical information (9). Derived from routine CBC data, neutrophil-to-lymphocyte ratio (NLR), platelet-to-lymphocyte ratio (PLR), and systemic immune-inflammation index (SII) have emerged as reliable, cost-effective inflammatory indicators for multiple inflammatory-related diseases, serving as promising auxiliary biomarkers for population-based prenatal research (10–12).

The preconception period is a critical window for safeguarding maternal physical health and establishing a favorable physiological basis for subsequent pregnancy outcomes. Abnormal elevation of maternal systemic inflammation during the preconception stage may disrupt endometrial microenvironmental stability, impair normal embryo implantation and subsequent placental development, and ultimately increase the susceptibility to adverse pregnancy outcomes. Adverse fetal and neonatal outcomes substantially raise the risks of intrapartum complications, metabolic dysfunction, and long-term cardiovascular diseases in both mothers and offspring, which severely compromise lifelong maternal and infant health (13–15). Therefore, targeted regulation of maternal inflammatory status before pregnancy is expected to mitigate the risk of adverse pregnancy-related events and reduce long-term health hazards for mothers and infants. Although numerous studies have explored the relationships between inflammatory status and pregnancy outcomes, most existing evidence focuses on gestational inflammation, while population-based studies specifically investigating preconception inflammatory levels and their association with adverse pregnancy outcomes remain limited. Against this research background, the present study aimed to systematically analyze the associations between preconception complete blood count-derived inflammatory indicators and specific adverse fetal, neonatal, and pregnancy outcomes.

## Methods

### Study design and study subjects

This study was based on data from the National Free Preconception Health Examination Project (NFPHEP). Funded by the National Health Commission and the Ministry of Finance of China, this nationwide population-based program was implemented in 220 counties and districts across 31 provincial-level administrative regions. The NFPHEP provides standardized free preconception physical examinations and professional health counseling for rural couples preparing for pregnancy within six months, and the detailed program design and implementation protocol have been described in previous studies (16–21). The present study enrolled 248,501 couples who achieved successful conception within six months after completing the standardized preconception screening from January 1, 2010, to December 31, 2012.

### Exclusion criteria

**Figure 1:**
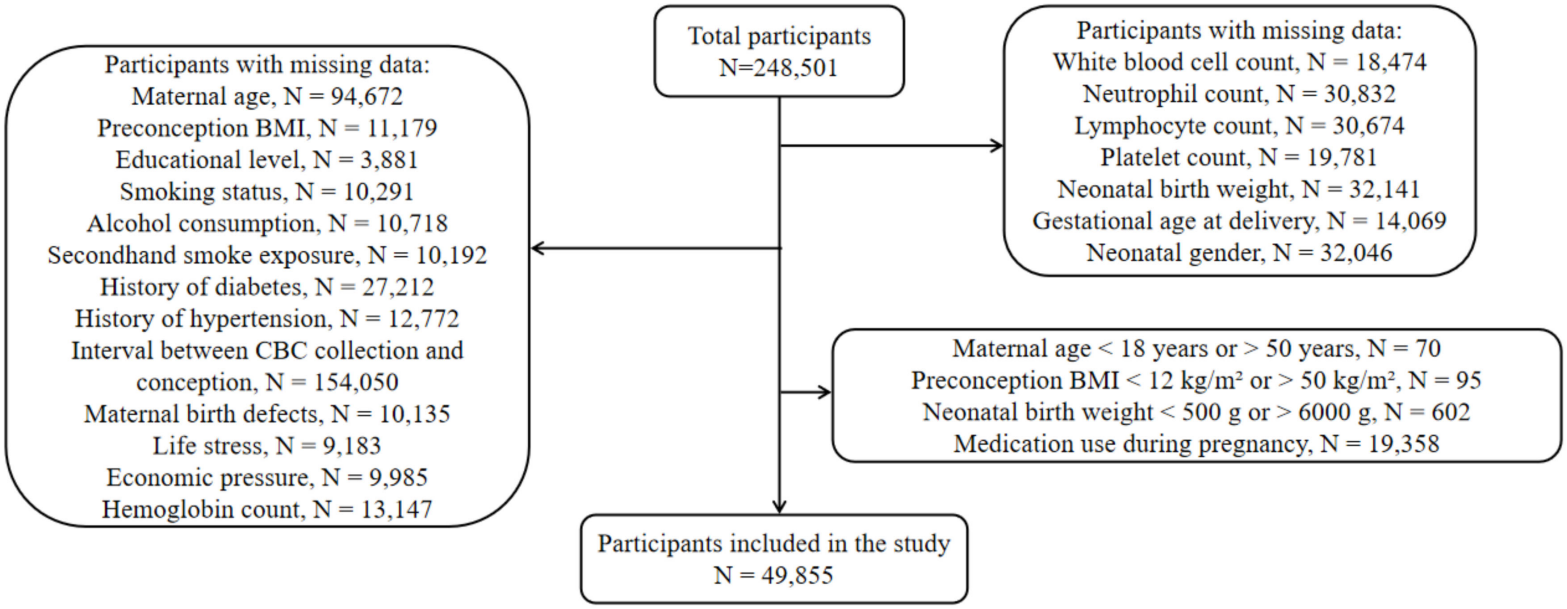
Flowchart of study participant selection. BMI, body mass index; CBC, complete blood count.

Participants who met any of the following conditions were excluded from the analysis: (1) missing data on core study indicators (CBC-derived inflammatory indicators), adverse fetal, neonatal and pregnancy outcomes, or key confounding covariates; (2) maternal age younger than 18 years or older than 50 years; (3) preconception body mass index (BMI) less than 12 kg/m² or greater than 50 kg/m²; (4) unknown neonatal gender; (5) maternal use of inflammation− or metabolism−affecting medications (e.g., glucocorticoids and immunosuppressants) before pregnancy, which may interfere with the stability of inflammatory indicators. Detailed inclusion and exclusion procedures for study participants are presented in **Figure 1**.

### Data collection

Uniformly trained investigators collected participants’ baseline data through face-to-face questionnaire interviews, including general demographic characteristics, living habits, family medical history, and personal medical history. Relevant laboratory blood test data and neonatal examination information were systematically recorded. All peripheral blood samples were analyzed by designated local laboratories in accordance with unified standard procedures, ensuring the accuracy and consistency of the test results.

### Detection and calculation of inflammatory indicators derived from complete blood count

We collected preconception CBC parameters of all female participants, including white blood cell count, neutrophil count, lymphocyte count, and platelet count. Three CBC-derived inflammatory indicators were calculated using the above laboratory results. The corresponding formulas are presented as follows (22, 23):

NLR = Neutrophil count ÷ Lymphocyte count
PLR = Platelet count ÷ Lymphocyte count
SII = (Platelet count × Neutrophil count) ÷ Lymphocyte count

### Definition of pregnancy outcomes

The pregnancy outcomes evaluated in this study included preterm birth, fetal birth defects, spontaneous abortion, stillbirth, and birth weight-related outcomes. Gestational age was defined as completed gestational weeks (e.g., 39 weeks + 6 days of pregnancy was counted as 39 gestational weeks). The specific definitions of each outcome were as follows:

Preterm birth: Delivery occurring at a gestational age of 28 weeks or more but less than 37 weeks (24);

Fetal birth defects: Structural, functional, or metabolic abnormalities occurring before birth, including anencephaly, spina bifida, encephalocele, congenital hydrocephalus, isolated cleft palate, cleft lip, cleft lip with cleft palate, microtia (including anotia), other external ear malformations (excluding microtia and anotia), esophageal atresia or stenosis, rectal and anal atresia or stenosis, hypospadias, bladder exstrophy, clubfoot, polydactyly, syndactyly, limb shortening malformations, congenital diaphragmatic hernia, omphalocele, gastroschisis, conjoined twins, Down syndrome (trisomy 21), and congenital heart disease (25);

Spontaneous abortion: Pregnancy termination caused by natural factors before 28 weeks of gestation (26);

Stillbirth: Fetal death at or after 28 weeks of gestation, with no vital signs such as breathing or heartbeat during intrauterine status or at delivery (27).

LBW: Neonatal birth weight less than 2500 g (28);

High birth weight (HBW): Neonatal birth weight greater than or equal to 4000 g (29);

Small for gestational age (SGA): Neonatal birth weight below the 10th percentile of birth weight for infants with the same gestational age and gender (30, 31);

LGA: Neonatal birth weight above the 90th percentile of birth weight for infants with the same gestational age and gender (30, 31).

### Covariates

Covariates included in the present study covered maternal age, preconception BMI, educational level (middle school or below/high school or above), history of chronic diseases (diabetes and hypertension; yes/no), smoking status (yes/no), second-hand smoke exposure (never/occasionally/frequently), alcohol consumption (never/occasionally/frequently), maternal birth defects (yes/no), anemia (yes/no), and thyroid disease (serving as a surrogate indicator for autoimmune diseases; yes/no), the interval between CBC testing and conception, life stress (yes/no), and economic pressure (yes/no). The diagnoses of hypertension and diabetes were comprehensively determined based on participants’ self-reported medical history and laboratory test results. In accordance with the Guidelines for the Prevention and Treatment of Diabetes in China (32), diabetes was defined as a fasting blood glucose (FBG) level ≥ 7.00 mmol/L or a previous clinical diagnosis of diabetes. Hypertension was defined as a systolic blood pressure (SBP) ≥ 140 mmHg (1 mmHg = 0.133 kPa) or diastolic blood pressure (DBP) ≥ 90 mmHg, or a previous clinical diagnosis of hypertension. Anemia was defined as a hemoglobin (Hb) concentration < 120 g/L based on routine blood test results (33).

### Statistical analysis

All data were analyzed using standard statistical methods. Continuous variables were presented as mean ± standard deviation or median (interquartile range) [M (P25, P75)] according to their distribution characteristics. The independent samples t-test or Mann-Whitney U test was used for between-group comparisons. Categorical variables were described as numbers (percentage) [n (%)], and the χ² test was adopted for group comparisons. Multivariate logistic regression analysis was performed to calculate the adjusted odds ratio (aOR) and 95% confidence interval (95% CI), so as to clarify the strength of associations between inflammatory indicators derived from CBC and adverse fetal, neonatal, and pregnancy outcomes. The restricted cubic spline (RCS) regression model was used to assess the dose-response relationships between log-transformed inflammatory indicators and the above adverse outcomes, and corresponding dose-response curves were plotted. The Benjamini-Hochberg false discovery rate (FDR) method was applied for multiple testing, with separate correction conducted for each outcome. All statistical analyses were implemented using Stata/MP 16.0 and R 4.3.1, and the significance level was set at α = 0.05.

To quantitatively assess the robustness of statistically significant associations against unmeasured confounding factors unavailable in our cohort database (including preconception infectious history, serum nutritional biomarkers, and paternal indicators), E-values were calculated for all associations with FDR-adjusted P < 0.05. The E-value is a well-established epidemiological sensitivity metric that only requires adjusted odds ratios and corresponding 95% confidence intervals from regression outputs, without raw data of missing confounders. It estimates the minimum confounding strength an unmeasured variable must exert to fully attenuate the detected significant associations between preconception inflammatory indicators and adverse fetal, neonatal, and pregnancy outcomes to the null. All E-value results are presented in **Supplementary Table 1**. Given the large sample size of this cohort, we simultaneously report adjusted OR values alongside P and FDR values throughout all results to avoid overinterpreting purely statistical significance without considering the practical magnitude of effect sizes.

This study used de-identified archived data from the NFPHEP, a national public health programme authorised by Chinese national health authorities in 2010 (official document: https://www.nhc.gov.cn/fys/c100078/201005/b6bfa324cc8c4720994d8e5861fc59b5.shtml). All participants provided written informed consent when enrolling in the original national screening programme in accordance with the official technical specifications of the project, and the present study was conducted in compliance with the Declaration of Helsinki principles.

## Results

### Baseline characteristics

A total of 49,855 study subjects were finally included in this study, among which 16,105 had adverse fetal, neonatal, and pregnancy outcomes, with an incidence rate of 32.30%. The overall baseline characteristics of the study subjects were as follows: the median age was 24.00 (22.00, 28.00) years, and the mean BMI was 21.03 ± 2.44 kg/m². Comparison between groups showed that the preconception BMI, educational level, hypertension history, alcohol consumption, second-hand smoke exposure, life stress, economic pressure, and NLR levels were significantly different between the adverse pregnancy outcome group and the non-adverse pregnancy outcome group (all P < 0.05).

Comparison of inflammatory indicators derived from preconception complete blood count between the two groups showed that the level of NLR in the adverse pregnancy outcome group was significantly different from that in the non-adverse pregnancy outcome group (P < 0.001), while there was no statistically significant difference in PLR and SII levels between the two groups (P = 0.165 and P = 0.963, respectively). Specifically, the NLR in the adverse pregnancy outcome group was 1.99 (1.61, 2.44), and that in the non-adverse pregnancy outcome group was 2.00 (1.63, 2.46) (P < 0.001); the PLR in the adverse pregnancy outcome group was 100.99 (78.57, 128.42), and that in the non-adverse pregnancy outcome group was 100.39 (78.12, 127.21) (P = 0.165); the SII in the adverse pregnancy outcome group was 393.31 (291.57, 521.88), and that in the non-adverse pregnancy outcome group was 392.33 (293.86, 517.67) (P = 0.963). The detailed baseline characteristics of the study subjects are shown in **Table 1**. Notably, all between-group differences here only reflect statistical significance, and small absolute group differences may achieve significant P-values due to the large overall sample size, which should be interpreted combined with actual clinical meaning rather than relying solely on P-values.

**Table 1:** Preconception baseline characteristics of participants.

Variables	Total (N=49,855)	Adverse fetal, neonatal, and pregnancy outcomes		P value
		Yes (N=16,105)	No (N=33,750)	
Age, years	24.00 (22.00, 28.00)	24.00 (22.00, 28.00)	24.00 (23.00, 28.00)	0.231
BMI, kg/m^2^	21.03 ± 2.44	21.12 ± 2.52	20.99 ± 2.40	<0.001
Interval between CBC collection and conception, days	62.00 (26.00, 122.00)	63.00 (26.00, 124.00)	62.00 (27.00, 122.00)	0.540
Maternal birth defects, No. (%)				0.639
Yes	112 (0.2)	39 (0.2)	73 (0.2)	
No	49,743 (99.8)	16,066 (99.8)	33,677 (99.8)	
Education level, No. (%)				0.008
Middle school or below	37,643 (75.5)	12,280 (76.2)	25,363 (75.1)	
High school or above	12,212 (24.5)	3,825 (23.8)	8,387 (24.9)	
Hypertension, No. (%)				0.030
Yes	1,704 (3.4)	592 (3.7)	1,112 (3.3)	
No	48,151 (96.6)	15,513 (96.3)	32,638 (96.7)	
Diabetes, No. (%)				0.315
Yes	685 (1.4)	234 (1.5)	451 (1.3)	
No	49,170 (98.6)	15,871 (98.5)	33,299 (98.7)	
Alcohol consumption, No. (%)				0.045
Never	48,082 (96.4)	15,573 (96.7)	32,509 (96.3)	
Occasionally	1,744 (3.5)	520 (3.2)	1,224 (3.6)	
Frequently	29 (0.1)	12 (0.1)	17 (0.1)	
Smoking status, No. (%)				1.000
Yes	178 (0.4)	57 (0.4)	121 (0.4)	
No	49,677 (99.6)	16,048 (99.6)	33,629 (99.6)	
Second-hand smoke exposure, No. (%)				<0.001
Never	39,764 (79.8)	12,553 (77.9)	27,211 (80.6)	
Occasionally	8,741 (17.5)	3,057 (19.0)	5,684 (16.8)	
Frequently	1,350 (2.7)	495 (3.1)	855 (2.5)	
Thyroid disease, No. (%)				0.405
Yes	46 (0.1)	18 (0.1)	28 (0.1)	
No	49,809 (99.9)	16,087 (99.9)	33,722 (99.9)	
Life stress, No. (%)				<0.001
Yes	5,690 (12.4)	1,667 (11.2)	4,023 (12.9)	
No	40,356 (87.6)	13,200 (88.8)	27,156 (87.1)	
Economic pressure, No. (%)				<0.001
Yes	5,552 (12.2)	1,675 (11.3)	3,877 (12.6)	
No	40,096 (87.8)	13,114 (88.7)	26,982 (87.4)	
Anemia, No. (%)				0.525
Yes	12,314 (24.7)	4,007 (24.9)	8,307 (24.6)	
No	37,541 (75.3)	12,098 (75.1)	25,443 (75.4)	
CBC-derived indicators				
NLR	2.00 (1.63, 2.45)	1.99 (1.61, 2.44)	2.00 (1.63, 2.46)	<0.001
PLR	100.55 (78.26, 127.63)	100.99 (78.57, 128.42)	100.39 (78.12, 127.21)	0.165
SII	392.67 (293.15, 519.03)	393.31 (291.57, 521.88)	392.33 (293.86, 517.67)	0.963

BMI, body mass index; CBC, complete blood count; NLR, neutrophil-to-lymphocyte ratio; PLR, platelet-to-lymphocyte ratio; SII, systemic immune-inflammation index.

Data are presented as mean ± standard deviation or median (interquartile range) for continuous variables, and n (%) for categorical variables.

### Association between inflammatory indicators derived from complete blood count and adverse fetal, neonatal, and pregnancy outcomes

As shown in **Table 2**, among 8,726 LGA cases, compared with the lowest quartile (Q1) of NLR, NLR Q2, Q3 and Q4 were correlated with lower LGA risk (Q2: OR = 0.83, 95% CI: 0.77–0.88, P < 0.001; Q3: OR = 0.82, 95% CI: 0.77–0.87, P < 0.001; Q4: OR = 0.85, 95% CI: 0.79–0.90, P < 0.001). For PLR, only the highest quartile (Q4, > 128.00) showed a correlation with elevated LGA risk (OR = 1.11, 95% CI: 1.04–1.19, P = 0.004), while PLR Q2 and Q3 showed no meaningful correlation with LGA risk. For SII, compared with Q1, SII Q2 and Q3 were linked to reduced LGA risk (Q2: OR = 0.91, 95% CI: 0.85–0.97, P = 0.009; Q3: OR = 0.92, 95% CI: 0.86–0.99, P = 0.023), and no meaningful correlation was detected between SII Q4 and LGA risk. Among 6,803 SGA cases, relative to NLR Q1, NLR Q2 and Q3 were associated with higher SGA risk (Q2: OR = 1.11, 95% CI: 1.03–1.19, P = 0.047; Q3: OR = 1.12, 95% CI: 1.04–1.21, P = 0.031), and NLR Q4 had no meaningful correlation with SGA risk. Neither PLR nor SII across all quartiles displayed meaningful correlations with SGA risk.

**Table 2:** Associations of CBC-derived inflammatory indicators with LGA and SGA.

	LGA (N= 8,726)		SGA (N=6,803)	
	OR(95% CI)	P value^*^	OR(95% CI)	P value^*^
NLR				
Q1(<1.63)	reference	–	reference	–
Q2(1.63-2.00)	0.83 (0.77, 0.88)	<0.001	1.11 (1.03, 1.19)	0.047
Q3(2.00-2.45)	0.82 (0.77, 0.87)	<0.001	1.12 (1.04, 1.21)	0.031
Q4(>2.45)	0.85 (0.79, 0.90)	<0.001	1.06 (0.98, 1.14)	0.429
PLR				
Q1(<78.30)	reference	–	reference	–
Q2(78.30-101.00)	1.03 (0.97, 1.10)	0.477	1.00 (0.93, 1.08)	0.924
Q3(101.00-128.00)	1.02 (0.96, 1.09)	0.597	1.04 (0.97, 1.12)	0.537
Q4(>128.00)	1.11 (1.04, 1.19)	0.004	0.98 (0.91, 1.06)	0.786
SII				
Q1(<293.00)	reference	–	reference	–
Q2(293.00-393.00)	0.91 (0.85, 0.97)	0.009	1.03 (0.95, 1.10)	0.744
Q3(393.00-519.00)	0.92 (0.86, 0.99)	0.023	1.05 (0.97, 1.13)	0.439
Q4(>519.00)	0.98 (0.92, 1.04)	0.587	1.05 (0.98, 1.13)	0.439

CBC, complete blood count; LGA, large for gestational age; SGA, small for gestational age; NLR, neutrophil-to-lymphocyte ratio; PLR, platelet-to-lymphocyte ratio; SII, systemic immune-inflammation index; OR, odds ratio.

^*^ P values were adjusted for multiple comparisons using the Benjamini-Hochberg false discovery rate (FDR) method, with correction applied separately for each outcome (LGA and SGA).

As shown in **Table 3**, among LBW infants (N=2,498), after adjusting for relevant confounding factors, no meaningful correlations were detected between all quartiles of NLR, PLR, or SII and LBW risk (all P > 0.05). Among HBW infants (N=3,726), compared with NLR Q1, only NLR Q4 (>2.45) correlated with lower HBW risk (OR=0.84, 95% CI: 0.77–0.93, P=0.005). No meaningful correlations were observed between any quartile of PLR or SII and HBW risk (all P > 0.05).

**Table 3:** Associations of CBC-derived inflammatory indicators with LBW and HBW.

	LBW (N= 2,498)		HBW (N=3,726)	
	OR(95% CI)	P value^*^	OR(95% CI)	P value^*^
NLR				
Q1(<1.63)	reference	–	reference	–
Q2(1.63-2.00)	0.99 (0.89, 1.11)	0.973	0.94 (0.86, 1.03)	0.292
Q3(2.00-2.45)	1.00 (0.89, 1.12)	0.973	0.91 (0.83, 1.00)	0.242
Q4(>2.45)	0.91 (0.81, 1.02)	0.787	0.84 (0.77, 0.93)	0.005
PLR				
Q1(<78.30)	reference	–	reference	–
Q2(78.30-101.00)	0.98 (0.87, 1.10)	0.973	1.07 (0.98, 1.18)	0.292
Q3(101.00-128.00)	0.95 (0.84, 1.06)	0.787	0.97 (0.88, 1.07)	0.632
Q4(>128.00)	0.93 (0.83, 1.04)	0.787	1.06 (0.97, 1.17)	0.292
SII				
Q1(<293.00)	reference	–	reference	–
Q2(293.00-393.00)	0.93 (0.83, 1.04)	0.787	0.94 (0.85, 1.03)	0.292
Q3(393.00-519.00)	0.94 (0.84, 1.05)	0.787	0.92 (0.83, 1.01)	0.242
Q4(>519.00)	0.98 (0.87, 1.09)	0.973	0.92 (0.84, 1.01)	0.242

Abbreviations: CBC, complete blood count; LBW, low birth weight; HBW, high birth weight; NLR, neutrophil-to-lymphocyte ratio; PLR, platelet-to-lymphocyte ratio; SII, systemic immune-inflammation index; OR, odds ratio.

^*^ P values were adjusted for multiple comparisons using the Benjamini-Hochberg false discovery rate (FDR) method, with correction applied separately for each outcome (LBW and HBW).

As shown in **Table 4**, among preterm birth cases (N=625), compared with NLR Q1, only NLR Q2 showed a correlation with reduced preterm birth risk (OR=0.69, 95% CI: 0.55–0.87, P=0.006), while NLR Q3 and Q4 showed no meaningful correlation with preterm birth risk. No meaningful correlations were observed between any quartile of PLR or SII and preterm birth risk (all P > 0.05). Among stillbirth cases (N=101), no meaningful correlations were observed between all quartiles of NLR, PLR, or SII and stillbirth risk (all P > 0.05).

**Table 4:** Associations of CBC-derived inflammatory indicators with preterm birth and stillbirth .

	Preterm birth (N=625)		Stillbirth (N=101)	
	OR(95% CI)	P value^*^	OR(95% CI)	P value^*^
NLR				
Q1(<1.63)	reference	–	reference	–
Q2(1.63-2.00)	0.69 (0.55, 0.87)	0.006	0.68 (0.38, 1.22)	0.853
Q3(2.00-2.45)	0.80 (0.64, 0.99)	0.122	1.04 (0.62, 1.76)	0.952
Q4(>2.45)	0.85 (0.68, 1.05)	0.264	0.90 (0.52, 1.55)	0.952
PLR				
Q1(<78.30)	reference	–	reference	–
Q2(78.30-101.00)	0.91 (0.73, 1.14)	0.487	1.04 (0.61, 1.79)	0.952
Q3(101.00-128.00)	0.87 (0.69, 1.09)	0.391	1.09 (0.64, 1.86)	0.952
Q4(>128.00)	1.06 (0.85, 1.32)	0.661	0.76 (0.42, 1.36)	0.853
SII				
Q1(<293.00)	reference	–	reference	–
Q2(293.00-393.00)	0.83 (0.66, 1.05)	0.264	1.37 (0.80, 2.36)	0.853
Q3(393.00-519.00)	0.90 (0.72, 1.13)	0.487	1.02 (0.57, 1.82)	0.952
Q4(>519.00)	1.10 (0.89, 1.36)	0.487	1.02 (0.58, 1.82)	0.952

CBC, complete blood count; NLR, neutrophil-to-lymphocyte ratio; PLR, platelet-to-lymphocyte ratio; SII, systemic immune-inflammation index; OR, odds ratio.

^*^ P values were adjusted for multiple comparisons using the Benjamini-Hochberg false discovery rate (FDR) method, with correction applied separately for each outcome (preterm birth and stillbirth).

As shown in **Supplementary Table 2**, among spontaneous abortion (N=42) and fetal birth defect (N=40) cases with extremely limited event volume, no statistically significant correlations were observed in the current analysis; however, such null findings should not be regarded as proof of a lack of underlying associations due to extremely low statistical power from sparse events.

### Non-linear relationship between CBC-derived inflammatory indicators and adverse fetal, neonatal, and pregnancy outcomes

**Figure 2:**
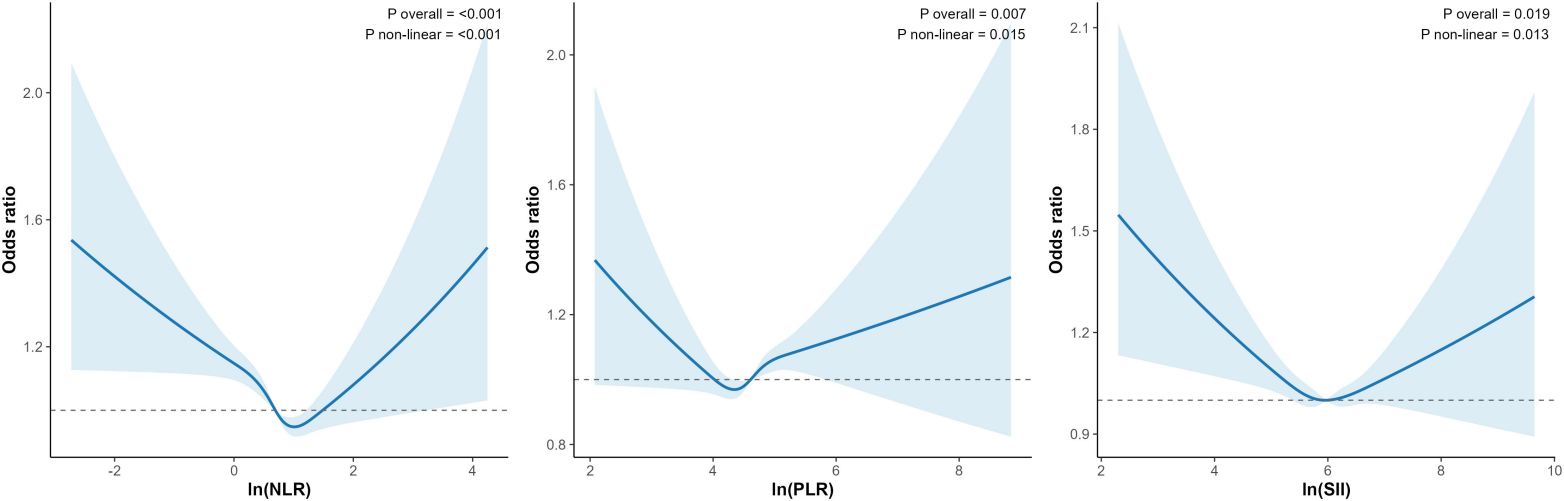
Nonlinear relationships between CBC-derived inflammatory indicators and large for gestational age. The restricted cubic spline regression models were fully adjusted for key confounding factors, including maternal age at pregnancy, preconception BMI, educational level, history of diabetes, history of hypertension, smoking status, alcohol consumption, second-hand smoke exposure, maternal birth defects, preconception thyroid disease (surrogate marker for autoimmune diseases), preconception anemia, life stress, and economic pressure. The horizontal dashed line denotes the reference odds ratio of 1.0, and the shaded area represents the 95% confidence interval of each fitted curves. CBC, complete blood count; NLR, neutrophil-to-lymphocyte ratio; PLR, platelet-to-lymphocyte ratio; SII, systemic immune-inflammation index; BMI, body mass index.

**Figure 3:**
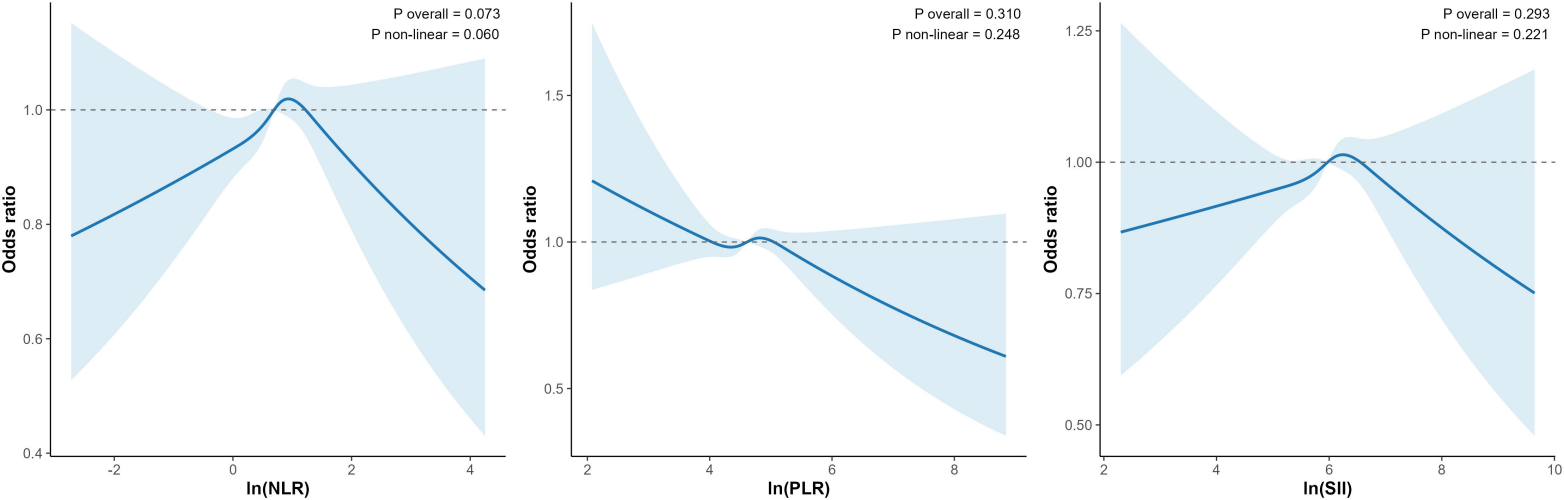
Nonlinear relationships between CBC-derived inflammatory indicators and small for gestational age. Restricted cubic spline regression models were fully adjusted for key confounding factors: maternal age at pregnancy, preconception BMI, educational level, history of diabetes, history of hypertension, smoking status, alcohol consumption, second-hand smoke exposure, maternal birth defects, preconception thyroid disease (surrogate marker for autoimmune diseases), preconception anemia, life stress, and economic pressure. The horizontal dashed line represents the reference odds ratio of 1.0, and the shaded areas indicate the 95% confidence intervals of the fitted curves. CBC, complete blood count; NLR, neutrophil-to-lymphocyte ratio; PLR, platelet-to-lymphocyte ratio; SII, systemic immune-inflammation index; BMI, body mass index.

**Figure 4:**
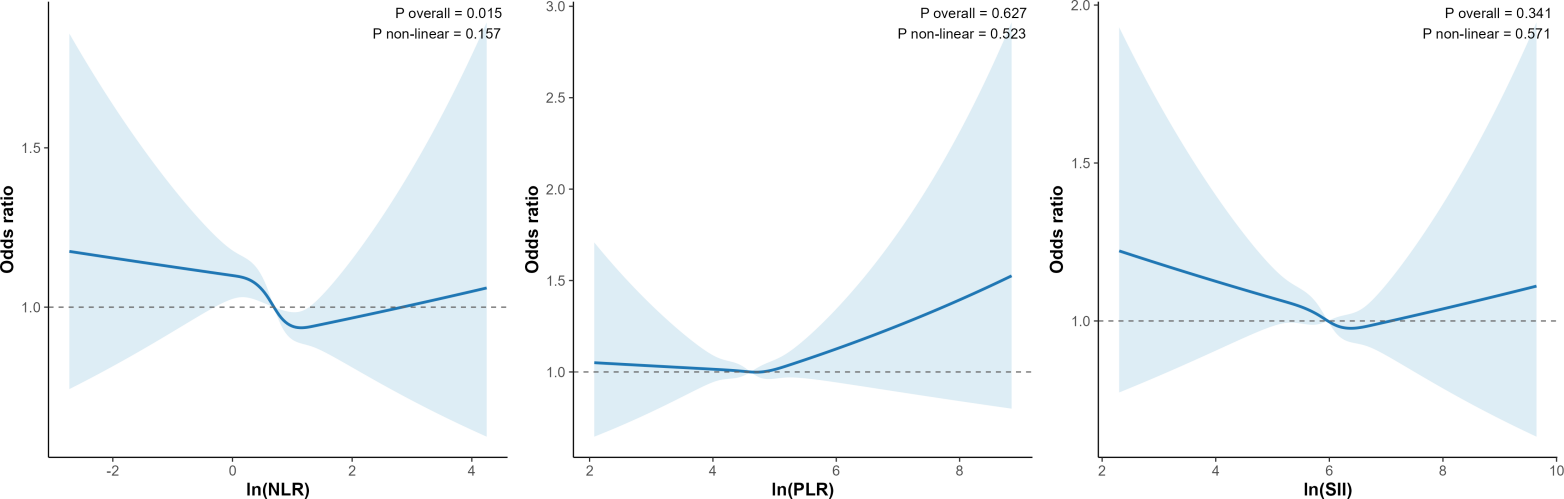
Nonlinear relationships between CBC-derived inflammatory indicators and high birth weight. Restricted cubic spline regression models were fully adjusted for key confounding factors: maternal age at pregnancy, preconception BMI, educational level, history of diabetes, history of hypertension, smoking status, alcohol consumption, second-hand smoke exposure, maternal birth defects, preconception thyroid disease (surrogate marker for autoimmune diseases), preconception anemia, life stress, and economic pressure. The horizontal dashed line represents the reference odds ratio of 1.0, and the shaded areas indicate the 95% confidence intervals of the fitted curves. CBC, complete blood count; NLR, neutrophil-to-lymphocyte ratio; PLR, platelet-to-lymphocyte ratio; SII, systemic immune-inflammation index; BMI, body mass index.

**Figure 5:**
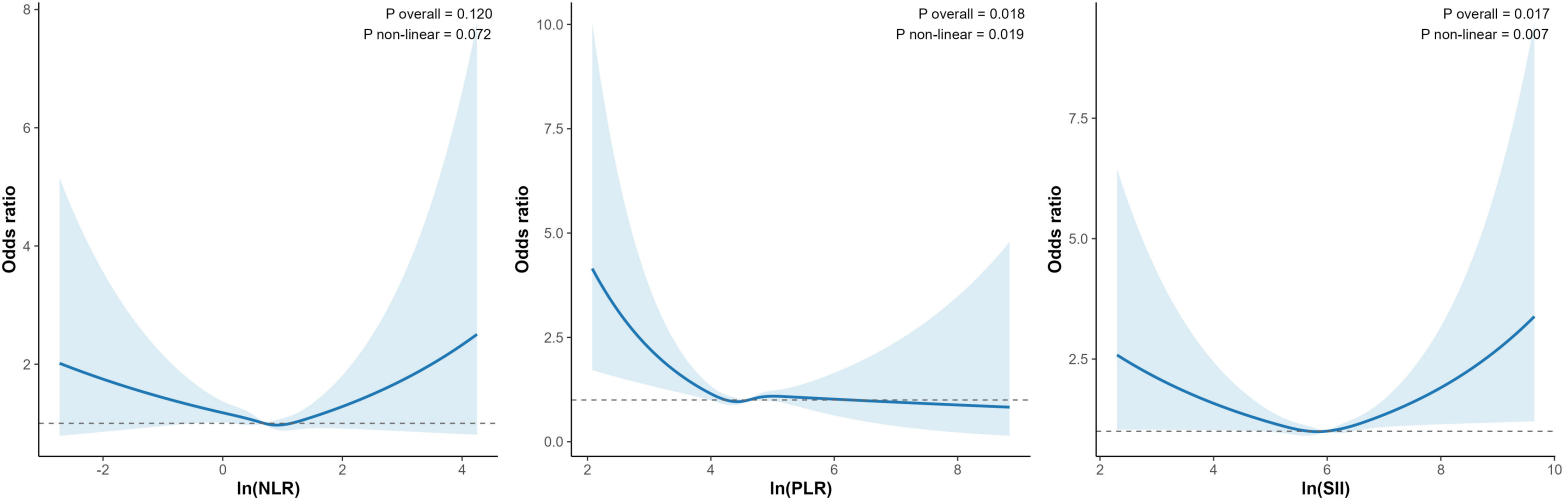
Nonlinear relationships between CBC-derived inflammatory indicators and preterm birth. Restricted cubic spline regression models were fully adjusted for key confounding factors: maternal age at pregnancy, preconception BMI, educational level, history of diabetes, history of hypertension, smoking status, alcohol consumption, second-hand smoke exposure, maternal birth defects, preconception thyroid disease (surrogate marker for autoimmune diseases), preconception anemia, life stress, and economic pressure. The horizontal dashed line represents the reference odds ratio of 1.0, and the shaded areas indicate the 95% confidence intervals of the fitted curves. CBC, complete blood count; NLR, neutrophil-to-lymphocyte ratio; PLR, platelet-to-lymphocyte ratio; SII, systemic immune-inflammation index; BMI, body mass index.

Restricted cubic spline regression analysis showed that NLR, PLR, and SII had distinct associations with different adverse fetal, neonatal, and pregnancy outcomes. All three indicators presented significant overall and non-linear relationships with LGA risk with U-shaped curves (**Figure 2**). No significant overall or non-linear associations of the three indicators were observed for SGA risk (**Figure 3**). For HBW risk, only NLR had a significant overall association without significant non-linearity, while PLR and SII showed no significant associations (**Figure 4**). For preterm birth, PLR and SII exhibited significant overall and non-linear associations with U-shaped trends, whereas NLR showed no significant associations (**Figure 5**). None of NLR, PLR, and SII were significantly correlated with LBW risk in either overall or non-linear analyses (**Supplementary Figure 1**). For spontaneous abortion, NLR and SII had significant overall associations but non-significant non-linear associations, while PLR showed no significant associations (**Supplementary Figure 2**). For fetal birth defects, only NLR had a significant overall association without significant non-linearity, and PLR showed no significant associations (**Supplementary Figure 3**).

## Discussion

Recent studies have shown that various maternal factors, such as preconception nutritional imbalance, abnormal placental function, maternal chronic diseases, preconception overweight and obesity, excessive weight gain during pregnancy, and multiparity, are closely related to adverse fetal, neonatal, and pregnancy outcomes. In addition, immune response and inflammatory balance play a key role in fetal growth regulation and have become a research focus in perinatal medicine (34, 35). This study explored the associations of preconception blood-derived inflammatory indicators with these adverse outcomes and identified distinct outcome specificity and threshold effects. Notably, although many associations reached low P-values due to the large sample size, the absolute magnitudes of effect sizes were modest, and clinical implications should not be overstated merely based on statistical significance. Quartile analysis indicated moderate NLR correlated with lower risks of LGA and preterm birth but higher SGA risk, the highest PLR increased LGA risk, and only elevated NLR reduced HBW risk; no indicators were associated with LBW, fetal birth defects, spontaneous abortion, or stillbirth. Restricted cubic spline regression further verified U-shaped non-linear relationships of all three indicators with LGA, and of PLR and SII with preterm birth. Overall, maternal inflammatory levels have complex threshold effects and outcome-specific associations rather than simple linear correlations with adverse fetal, neonatal, and pregnancy outcomes, and these observed correlations cannot be interpreted as fully independent links due to unmeasured shared confounders.

Complete blood count is one of the most widely used and convenient basic detection items in clinical practice, which can indirectly reflect the systemic inflammatory status via blood cell parameters (36, 37). This study found that preconception levels of white blood cells, neutrophils, lymphocytes, and platelets were significantly correlated with adverse fetal, neonatal, and pregnancy outcomes, which is in line with findings from previous studies. For example, Zhang et al. conducted a cohort study involving 24,143 Chinese pregnant women and found that elevated white blood cell counts were linked to higher risks of preterm birth and low birth weight (38). Sun et al. reported that elevated neutrophil counts in the first trimester were associated with macrosomia (39). Multiple studies by Christoforaki V, Levy O, Akgun N, and other researchers further demonstrated that NLR and related inflammatory indices were associated with adverse pregnancy outcomes, including spontaneous abortion, SGA, and preterm birth (40–42). Collectively, these findings support our results and indicate that abnormal levels of inflammatory markers such as NLR, PLR, and SII are closely related to aberrant fetal growth and development.

In addition, this study further found that preconception NLR, PLR, and SII exhibited distinct outcome-specific and threshold-dependent associations with adverse fetal, neonatal, and pregnancy outcomes. Most previous studies have demonstrated that elevated inflammatory indicators act as risk factors for adverse pregnancy outcomes (43, 44). However, the present study identified non-monotonic U-shaped nonlinear associations of NLR, PLR, and SII with LGA, as well as U-shaped relationships of PLR and SII with preterm birth. Stratified analyses revealed that moderate levels of NLR and SII exerted protective effects against LGA, while high PLR levels increased the risk of LGA; moderate NLR levels reduced the risk of preterm birth. In contrast, medium NLR levels were associated with an elevated risk of SGA, and only high NLR levels decreased the risk of HBW. Notably, no significant quartile-based associations were observed between the three inflammatory indicators and LBW, fetal birth defects, spontaneous abortion, or stillbirth in this study, which was inconsistent with previous studies that confirmed significant correlations between inflammatory abnormalities and these adverse outcomes (45, 46). The discrepancies may be explained by several key factors: most prior studies focused on gestational inflammatory levels, whereas the current study specifically targeted preconception inflammatory indicators. In addition, the relatively small sample size of rare adverse outcomes and population heterogeneity in this study may contribute to the inconsistent results. The specific underlying mechanisms remain to be further explored.

At the same time, accumulating clinical evidence indicates that positive lifestyle interventions, including regular exercise and dietary adjustment, can effectively restore maternal immune-inflammatory balance and alleviate systemic inflammation, thereby ameliorating inflammation-related adverse pregnancy risks and optimizing maternal and infant outcomes (47, 48). Combined with existing clinical evidence and the present findings, the readily available preconception inflammatory markers identified in this study provide novel and practical evidence for targeted preconception inflammatory intervention and the prevention of adverse fetal, neonatal, and pregnancy outcomes. Based on a rural Chinese pregnant women cohort recruited from 2010 to 2012, this study not only reveals the relationships between preconception immune-inflammatory status and adverse pregnancy and fetal outcomes, but also offers population-specific clinical insights and theoretical references. Given the single-center and rural population limitation of this cohort, the generalizability of these findings to contemporary urban populations or other ethnic groups remains to be further validated by updated real-world data.

This study has the following strengths and innovations. First, this large-scale prospective population cohort covered 31 provinces across northern and southern China. The complete baseline information of participants and rigorous follow-up data regarding adverse fetal, neonatal, and pregnancy outcomes effectively reduced selection and information bias, thereby substantially improving the validity and reliability of the research findings. Second, this study is the first to systematically explore the complex associations between preconception CBC-derived inflammatory indicators and adverse fetal, neonatal, and pregnancy outcomes from the perspective of immune-inflammatory balance. This study clarified the magnitude of these associations and provides a novel research basis for understanding the influence of preconception immune-inflammatory status on fetal and pregnancy outcomes. Third, complete blood count testing is economical, convenient, and highly accessible without the requirement for specialized equipment or professional techniques. It is suitable for large-scale population epidemiological investigations and clinical risk assessment in similar rural populations, and has the potential to serve as practical biomarkers for identifying high-risk groups for adverse fetal, neonatal, and pregnancy outcomes.

This study has several limitations that need to be improved in future research. First, all participants were rural Chinese women enrolled in the National Free Preconception Health Examination Program between 2010 and 2012. Over the past decade, dramatic changes in population lifestyle, dietary nutrition, and clinical diagnosis and treatment modes have reduced the timeliness of these historical data. In addition, this study only recruited participants from rural areas with specific regional and population characteristics, limiting the generalizability of the findings to urban populations, other ethnic groups, and contemporary pregnant cohorts.

Second, although multiple confounding factors, including maternal age, preconception BMI, chronic disease history, and anemia, were adjusted in this study, several confounding variables could not be controlled for. Specifically, detailed data on other autoimmune diseases were not collected, and only thyroid disease was adopted as a proxy indicator for immune-related disorders. Meanwhile, baseline information regarding preconception infection history, nutritional status (serum folate, iron, and vitamin D levels), sleep status, gestational weight gain, and paternal-related indicators was unavailable and could not be adjusted in the analysis. We further systematically analyzed the potential confounding bias introduced by these unmeasured covariates. First, undiagnosed preconception or prenatal infections simultaneously elevate systemic inflammatory levels and independently increase the risk of adverse fetal, neonatal, and pregnancy outcomes, generating positive confounding that slightly overestimates the true association magnitudes (49, 50). Second, deficiencies in folate, iron, or vitamin D aggravate maternal inflammation and impair fetal development synchronously, which also creates mild positive confounding (51, 52). Third, paternal inflammatory dysfunction and infertility correlate with maternal inflammatory profiles and adverse fetal, neonatal, and pregnancy outcomes; the absence of paternal data may further amplify this positive bias (53, 54). To quantitatively test how robust our findings are to such unmeasured confounding, we calculated E-values for all FDR-significant associations. The E-value for the association between NLR-2 and preterm birth reached 2.238, indicating moderate robustness against unmeasured confounding; E-values for other significant associations ranged from 1.388 to 1.748, suggesting that mild-to-moderate unmeasured confounding would be required to invalidate these findings. Even though multiple outcomes showed associations with P < 0.001 in this large sample, the missing key confounders described above may still compromise the true effect sizes, and replication in cohorts with complete covariate data is necessary.

Third, all inflammatory indicators were calculated based on a single preconception complete blood count test result. The time interval between blood sampling and conception varied among individuals, and a single detection failed to reflect the dynamic fluctuations of inflammatory levels around conception and during pregnancy, which may lead to exposure misclassification and measurement bias. Future longitudinal cohort studies with repeated inflammatory indicator detections are warranted to comprehensively and dynamically clarify the associations between inflammatory indicators and adverse pregnancy outcomes.

Fourth, hypertension and diabetes were diagnosed through a combination of participants’ self-reported medical histories and objective laboratory test results. Prior studies have demonstrated that a considerable proportion of participants lack adequate awareness of their chronic conditions, and self-reported information alone tends to underestimate the true prevalence of hypertension and inevitably introduces recall bias (55). Although laboratory blood indicators were incorporated to mitigate this drawback, this mixed subjective-objective diagnostic framework still cannot fully eliminate misclassification of chronic disease status, which may slightly reduce the adjustment efficacy of these two key confounding variables.

Fifth, analyses of fetal birth defects (40 events) and spontaneous abortion (42 events) were restricted by extremely limited case numbers, which brings classic rare event bias and insufficient statistical power to multivariate logistic regression. Readers should interpret the non-significant results of these two outcomes with extreme caution. Sparse events easily generate unstable odds ratios and wide confidence intervals, so we cannot draw any definitive inference that there is no true correlation between inflammatory indicators and the two adverse events merely based on the current dataset.

Finally, restricted by the original cohort data, this study only focused on fetal and neonatal adverse outcomes such as SGA, LGA, and LBW, without including common maternal pregnancy complications, including gestational hypertension, preeclampsia, gestational diabetes mellitus, placental abruption, and postpartum hemorrhage.

## Conclusion

In conclusion, based on the large-scale prospective cohort data of rural women of childbearing age recruited from 2010 to 2012, this study systematically analyzed the associations between preconception CBC-derived inflammatory indicators and adverse fetal and neonatal outcomes. After adjusting all available covariates, we only identified statistical correlations rather than fully independent links between these inflammatory markers and adverse outcomes, with obvious outcome-specific and non-linear threshold characteristics. It should be highlighted that our large sample size enables small effect sizes to reach statistically significant P-values, so the clinical practical value of these weak correlations should not be overstated. At present, there are no unified clinically recognized cutoff values for preconception inflammatory indicators. The association ranges obtained in this study are only statistical results specific to this cohort and can provide reference data for subsequent similar research. Large-sample, multicenter prospective cohorts based on contemporary populations are required to externally validate our findings, and further research on targeted preconception inflammatory intervention strategies will help optimize perinatal health management and generate solid clinical evidence for maternal and infant care.

## Data Availability

The datasets presented in this article are not readily available because the data underlying this article cannot be shared publicly due to the privacy of individuals who participated in the study. The data will be shared on a reasonable request to the corresponding authors. Requests to access the datasets should be directed to Hui Pan, E-mail: panhui20111111@163.com.
