# Supplementary material for: Association between inflammatory indicators derived from preconception complete blood count and specific adverse fetal, neonatal, and pregnancy outcomes: a national cohort study
Source: Front Endocrinol (Lausanne). 2026 Jul 22;17:1886578. doi: 10.3389/fendo.2026.1886578 (PMC13437505; doi:10.3389/fendo.2026.1886578)
Supplement: Supplementary file 1 [file SupplementaryFile1.docx]

Supplement Figure 1. Nonlinear relationships between CBC-derived inflammatory indicators and low birth weight

Note: Restricted cubic spline regression models were fully adjusted for key confounding factors: maternal age at pregnancy, preconception BMI, educational level, history of diabetes, history of hypertension, smoking status, alcohol consumption, second-hand smoke exposure, maternal birth defects, preconception thyroid disease (surrogate marker for autoimmune diseases), preconception anemia, life stress, and economic pressure. The horizontal dashed line represents the reference odds ratio of 1.0, and the shaded areas indicate the 95% confidence intervals of the fitted curves.

Abbreviations: CBC, complete blood count; NLR, neutrophil-to-lymphocyte ratio; PLR, platelet-to-lymphocyte ratio; SII, systemic immune-inflammation index; BMI, body mass index

Supplement Figure 2. Nonlinear relationships between CBC-derived inflammatory indicators and spontaneous abortion

Note: Restricted cubic spline regression models were fully adjusted for key confounding factors: maternal age at pregnancy, preconception BMI, educational level, history of diabetes, history of hypertension, smoking status, alcohol consumption, second-hand smoke exposure, maternal birth defects, preconception thyroid disease (surrogate marker for autoimmune diseases), preconception anemia, life stress, and economic pressure. The horizontal dashed line represents the reference odds ratio of 1.0, and the shaded areas indicate the 95% confidence intervals of the fitted curves.

Abbreviations: CBC, complete blood count; NLR, neutrophil-to-lymphocyte ratio; PLR, platelet-to-lymphocyte ratio; SII, systemic immune-inflammation index; BMI, body mass index

Supplement Figure 3. Nonlinear relationships between CBC-derived inflammatory indicators and fetal birth defects

Note: Restricted cubic spline regression models were fully adjusted for key confounding factors: maternal age at pregnancy, preconception BMI, educational level, history of diabetes, history of hypertension, smoking status, alcohol consumption, second-hand smoke exposure, maternal birth defects, preconception thyroid disease (surrogate marker for autoimmune diseases), preconception anemia, life stress, and economic pressure. The horizontal dashed line represents the reference odds ratio of 1.0, and the shaded areas indicate the 95% confidence intervals of the fitted curves.

Abbreviations: CBC, complete blood count; NLR, neutrophil-to-lymphocyte ratio; PLR, platelet-to-lymphocyte ratio; BMI, body mass index

Supplementary Table 1. E-values for associations between preconception inflammatory indicators and adverse fetal, neonatal, and pregnancy outcomes

| exposure | outcome | event_n | term | OR | CI | P | FDR_by_outcome | E-value |
| --- | --- | --- | --- | --- | --- | --- | --- | --- |
| NLR | Preterm birth | 625 | NLR-2 | 0.694 | 0.554-0.87 | 0.001 | 0.006 | 2.238 |
| NLR | Preterm birth | 625 | NLR-3 | 0.797 | 0.641-0.99 | 0.041 | 0.122 | 1.820 |
| NLR | Preterm birth | 625 | NLR-4 | 0.848 | 0.684-1.051 | 0.132 | 0.264 | 1.639 |
| PLR | Preterm birth | 625 | PLR-2 | 0.909 | 0.726-1.138 | 0.406 | 0.487 | 1.432 |
| PLR | Preterm birth | 625 | PLR-3 | 0.87 | 0.693-1.092 | 0.228 | 0.391 | 1.564 |
| PLR | Preterm birth | 625 | PLR-4 | 1.059 | 0.852-1.315 | 0.606 | 0.661 | 1.309 |
| SII | Preterm birth | 625 | SII-2 | 0.834 | 0.663-1.049 | 0.121 | 0.264 | 1.688 |
| SII | Preterm birth | 625 | SII-3 | 0.901 | 0.72-1.128 | 0.365 | 0.487 | 1.459 |
| SII | Preterm birth | 625 | SII-4 | 1.096 | 0.885-1.358 | 0.4 | 0.487 | 1.420 |
| NLR | Fetal birth defects | 40 | NLR-2 | 0.371 | 0.156-0.887 | 0.026 | 0.103 | 4.833 |
| NLR | Fetal birth defects | 40 | NLR-3 | 0.326 | 0.129-0.822 | 0.018 | 0.103 | 5.586 |
| NLR | Fetal birth defects | 40 | NLR-4 | 0.415 | 0.18-0.955 | 0.039 | 0.116 | 4.253 |
| PLR | Fetal birth defects | 40 | PLR-2 | 1.012 | 0.401-2.554 | 0.98 | 0.98 | 1.122 |
| PLR | Fetal birth defects | 40 | PLR-3 | 0.798 | 0.296-2.147 | 0.655 | 0.873 | 1.816 |
| PLR | Fetal birth defects | 40 | PLR-4 | 1.716 | 0.749-3.934 | 0.202 | 0.404 | 2.824 |
| SII | Fetal birth defects | 40 | SII-2 | 0.876 | 0.404-1.898 | 0.737 | 0.885 | 1.544 |
| SII | Fetal birth defects | 40 | SII-3 | 0.368 | 0.132-1.023 | 0.055 | 0.133 | 4.878 |
| SII | Fetal birth defects | 40 | SII-4 | 0.655 | 0.283-1.516 | 0.323 | 0.485 | 2.423 |
| NLR | Spontaneous abortion | 42 | NLR-2 | 1.269 | 0.555-2.903 | 0.572 | 0.833 | 1.853 |
| NLR | Spontaneous abortion | 42 | NLR-3 | 1.424 | 0.635-3.189 | 0.391 | 0.833 | 2.201 |
| NLR | Spontaneous abortion | 42 | NLR-4 | 0.392 | 0.122-1.256 | 0.115 | 0.445 | 4.540 |
| PLR | Spontaneous abortion | 42 | PLR-2 | 0.863 | 0.41-1.819 | 0.699 | 0.833 | 1.588 |
| PLR | Spontaneous abortion | 42 | PLR-3 | 0.53 | 0.224-1.254 | 0.148 | 0.445 | 3.180 |
| PLR | Spontaneous abortion | 42 | PLR-4 | 0.402 | 0.155-1.038 | 0.06 | 0.359 | 4.411 |
| SII | Spontaneous abortion | 42 | SII-2 | 0.772 | 0.35-1.704 | 0.522 | 0.833 | 1.914 |
| SII | Spontaneous abortion | 42 | SII-3 | 0.824 | 0.38-1.79 | 0.625 | 0.833 | 1.723 |
| SII | Spontaneous abortion | 42 | SII-4 | 0.347 | 0.125-0.965 | 0.042 | 0.359 | 5.211 |
| NLR | Stillbirth | 101 | NLR-2 | 0.677 | 0.377-1.215 | 0.191 | 0.853 | 2.317 |
| NLR | Stillbirth | 101 | NLR-3 | 1.043 | 0.617-1.761 | 0.875 | 0.952 | 1.255 |
| NLR | Stillbirth | 101 | NLR-4 | 0.901 | 0.523-1.552 | 0.707 | 0.952 | 1.459 |
| PLR | Stillbirth | 101 | PLR-2 | 1.042 | 0.606-1.791 | 0.881 | 0.952 | 1.251 |
| PLR | Stillbirth | 101 | PLR-3 | 1.09 | 0.637-1.864 | 0.753 | 0.952 | 1.403 |
| PLR | Stillbirth | 101 | PLR-4 | 0.759 | 0.422-1.363 | 0.356 | 0.853 | 1.964 |
| SII | Stillbirth | 101 | SII-2 | 1.373 | 0.799-2.359 | 0.251 | 0.853 | 2.089 |
| SII | Stillbirth | 101 | SII-3 | 1.018 | 0.569-1.821 | 0.952 | 0.952 | 1.153 |
| SII | Stillbirth | 101 | SII-4 | 1.024 | 0.576-1.819 | 0.937 | 0.952 | 1.181 |
| NLR | LBW | 2498 | NLR-2 | 0.993 | 0.887-1.112 | 0.909 | 0.973 | 1.091 |
| NLR | LBW | 2498 | NLR-3 | 0.998 | 0.891-1.118 | 0.973 | 0.973 | 1.047 |
| NLR | LBW | 2498 | NLR-4 | 0.906 | 0.807-1.017 | 0.094 | 0.787 | 1.442 |
| PLR | LBW | 2498 | PLR-2 | 0.979 | 0.874-1.095 | 0.706 | 0.973 | 1.169 |
| PLR | LBW | 2498 | PLR-3 | 0.945 | 0.843-1.059 | 0.328 | 0.787 | 1.306 |
| PLR | LBW | 2498 | PLR-4 | 0.929 | 0.829-1.041 | 0.205 | 0.787 | 1.363 |
| SII | LBW | 2498 | SII-2 | 0.928 | 0.828-1.04 | 0.197 | 0.787 | 1.367 |
| SII | LBW | 2498 | SII-3 | 0.939 | 0.838-1.052 | 0.281 | 0.787 | 1.328 |
| SII | LBW | 2498 | SII-4 | 0.976 | 0.872-1.092 | 0.67 | 0.973 | 1.183 |
| NLR | HBW | 3726 | NLR-2 | 0.942 | 0.859-1.033 | 0.206 | 0.292 | 1.317 |
| NLR | HBW | 3726 | NLR-3 | 0.912 | 0.83-1.002 | 0.056 | 0.242 | 1.422 |
| NLR | HBW | 3726 | NLR-4 | 0.843 | 0.766-0.927 | <0.001 | 0.005 | 1.656 |
| PLR | HBW | 3726 | PLR-2 | 1.074 | 0.977-1.18 | 0.138 | 0.292 | 1.356 |
| PLR | HBW | 3726 | PLR-3 | 0.969 | 0.88-1.067 | 0.527 | 0.632 | 1.214 |
| PLR | HBW | 3726 | PLR-4 | 1.061 | 0.965-1.166 | 0.219 | 0.292 | 1.315 |
| SII | HBW | 3726 | SII-2 | 0.938 | 0.854-1.029 | 0.177 | 0.292 | 1.332 |
| SII | HBW | 3726 | SII-3 | 0.915 | 0.833-1.005 | 0.065 | 0.242 | 1.412 |
| SII | HBW | 3726 | SII-4 | 0.92 | 0.837-1.01 | 0.081 | 0.242 | 1.394 |
| NLR | SGA | 6803 | NLR-2 | 1.105 | 1.027-1.189 | 0.008 | 0.047 | 1.446 |
| NLR | SGA | 6803 | NLR-3 | 1.119 | 1.04-1.205 | 0.003 | 0.031 | 1.484 |
| NLR | SGA | 6803 | NLR-4 | 1.057 | 0.981-1.138 | 0.143 | 0.429 | 1.302 |
| PLR | SGA | 6803 | PLR-2 | 1.004 | 0.933-1.079 | 0.924 | 0.924 | 1.067 |
| PLR | SGA | 6803 | PLR-3 | 1.038 | 0.966-1.116 | 0.313 | 0.537 | 1.237 |
| PLR | SGA | 6803 | PLR-4 | 0.981 | 0.912-1.055 | 0.6 | 0.786 | 1.160 |
| SII | SGA | 6803 | SII-2 | 1.026 | 0.953-1.103 | 0.496 | 0.744 | 1.189 |
| SII | SGA | 6803 | SII-3 | 1.047 | 0.973-1.126 | 0.219 | 0.439 | 1.269 |
| SII | SGA | 6803 | SII-4 | 1.051 | 0.977-1.13 | 0.183 | 0.439 | 1.283 |
| NLR | LGA | 8726 | NLR-2 | 0.826 | 0.774-0.881 | <0.001 | <0.001 | 1.716 |
| NLR | LGA | 8726 | NLR-3 | 0.817 | 0.765-0.872 | <0.001 | <0.001 | 1.748 |
| NLR | LGA | 8726 | NLR-4 | 0.845 | 0.792-0.902 | <0.001 | <0.001 | 1.649 |
| PLR | LGA | 8726 | PLR-2 | 1.031 | 0.965-1.102 | 0.358 | 0.477 | 1.210 |
| PLR | LGA | 8726 | PLR-3 | 1.021 | 0.955-1.091 | 0.547 | 0.597 | 1.167 |
| PLR | LGA | 8726 | PLR-4 | 1.11 | 1.04-1.185 | 0.002 | 0.004 | 1.459 |
| SII | LGA | 8726 | SII-2 | 0.91 | 0.853-0.972 | 0.005 | 0.009 | 1.429 |
| SII | LGA | 8726 | SII-3 | 0.922 | 0.864-0.985 | 0.015 | 0.023 | 1.388 |
| SII | LGA | 8726 | SII-4 | 0.977 | 0.916-1.043 | 0.489 | 0.587 | 1.179 |

Supplementary Table 2. Associations of CBC-derived inflammatory indicators with spontaneous abortion and fetal birth defects

|  | Spontaneous abortion (N=42) | | Fetal birth defects (N=40) | |
| --- | --- | --- | --- | --- |
|  | OR(95% CI) | P value^*^ | OR(95% CI) | P value^*^ |
| NLR | | | | |
| Q1(＜1.63) | reference | - | reference | - |
| Q2(1.63-2.00) | 1.27 (0.56, 2.90) | 0.833 | 0.37 (0.16, 0.89) | 0.103 |
| Q3(2.00-2.45) | 1.42 (0.64, 3.19) | 0.833 | 0.33 (0.13, 0.82) | 0.103 |
| Q4(＞2.45) | 0.39 (0.12, 1.26) | 0.445 | 0.42 (0.18, 0.96) | 0.116 |
| PLR | | | | |
| Q1(＜78.30) | reference | - | reference | - |
| Q2(78.30-101.00) | 0.86 (0.41, 1.82) | 0.833 | 1.01 (0.40, 2.55) | 0.980 |
| Q3(101.00-128.00) | 0.53 (0.22, 1.25) | 0.445 | 0.80 (0.30, 2.15) | 0.873 |
| Q4(＞128.00) | 0.40 (0.16, 1.04) | 0.359 | 1.72 (0.75, 3.93) | 0.404 |
| SII | | | | |
| Q1(＜293.00) | reference | - | reference | - |
| Q2(293.00-393.00) | 0.77 (0.35, 1.70) | 0.833 | 0.88 (0.40, 1.90) | 0.885 |
| Q3(393.00-519.00) | 0.82 (0.38, 1.79) | 0.833 | 0.37 (0.13, 1.02) | 0.133 |
| Q4(＞519.00) | 0.35 (0.13, 0.97) | 0.359 | 0.66 (0.28, 1.52) | 0.485 |

Abbreviations: CBC, complete blood count; NLR, neutrophil-to-lymphocyte ratio; PLR, platelet-to-lymphocyte ratio; SII, systemic immune-inflammation index; OR, odds ratio

^*^P values were adjusted for multiple comparisons using the Benjamini-Hochberg false discovery rate (FDR) method, with correction applied separately for each outcome (spontaneous abortion and fetal birth defects).
